# Effect of voxel size in cone-beam computed tomography on surface area measurements of dehiscences and fenestrations in the lower anterior buccal region

**DOI:** 10.1007/s00784-022-04521-x

**Published:** 2022-05-05

**Authors:** B. J. van Leeuwen, P. U. Dijkstra, J. A. Dieters, H. P. J. Verbeek, A. M. Kuijpers-Jagtman, Y. Ren

**Affiliations:** 1 Department of Orthodontics, University Medical Center Groningen, University of Groningen, Hanzeplein 1, 9713 Groningen, GZ Netherlands; 2 Department of Rehabilitation and Department of Oral and Maxillofacial Surgery, University Medical Center Groningen, University of Groningen, Hanzeplein 1, 9713 Groningen, GZ Netherlands; 3 Department of Orthodontics and Dentofacial Orthopedics, School of Dental Medicine/Medical Faculty, University of Bern, Freiburgstrasse 7, 3010 Bern, CH Switzerland; 4 Faculty of Dentistry, Universitas Indonesia, Campus Salemba, Jalan Salemba Raya No. 4, Jakarta, 10430 Indonesia

**Keywords:** Cone-beam computed tomography, Dehiscence, Fenestration, Accuracy, Reliability

## Abstract

**Objectives:**

This study aims to assess whether different voxel sizes in cone-beam computed tomography (CBCT) affected surface area measurements of dehiscences and fenestrations in the mandibular anterior buccal region.

**Materials and methods:**

Nineteen dry human mandibles were scanned with a surface scanner (SS). Wax was attached to the mandibles as a soft tissue equivalent. Three-dimensional digital models were generated with a CBCT unit, with voxel sizes of 0.200 mm (VS200), 0.400 mm (VS400), and 0.600 mm (VS600). The buccal surface areas of the six anterior teeth were measured (in mm^2^) to evaluate areas of dehiscences and fenestrations. Differences between the CBCT and SS measurements were determined in a linear mixed model analysis.

**Results:**

The mean surface area per tooth was 88.3 ± 24.0 mm^2^, with the SS, and 94.6 ± 26.5 (VS200), 95.1 ± 27.3 (VS400), and 96.0 ± 26.5 (VS600), with CBCT scans. Larger surface areas resulted in larger differences between CBCT and SS measurements (− 0.1 β, SE = 0.02, *p* < 0.001). Deviations from SS measurements were larger with VS600, compared to VS200 (1.3 β, SE = 0.05, *P* = 0.009). Fenestrations were undetectable with CBCT.

**Conclusions:**

CBCT imaging magnified the surface area of dehiscences in the anterior buccal region of the mandible by 7 to 9%. The larger the voxel size, the larger the deviation from SS measurements. Fenestrations were not detectable with CBCT.

**Clinical relevance:**

CBCT is an acceptable tool for measuring dehiscences but not fenestrations. However, CBCT overestimates the size of dehiscences, and the degree of overestimation depends on the actual dehiscence size and CBCT voxel size employed.

## Introduction

The positions of the mandibular anterior teeth play an important role in orthodontic treatment planning, due to the restricted anatomic space in the symphysis. Excessive sagittal movement, tipping, and a narrow symphysis may result in bony dehiscences and fenestrations [1–3]. The presence and amount of bone in the anterior buccal mandibular region are often unknown. An undiagnosed buccal bony defect could potentially cause a gingival recession, which results in a compromised esthetic treatment outcome [4–6]. Bony defects, particularly dehiscences, are common in the anterior buccal region of the mandible, regardless of whether the subject has received orthodontic treatment [7, 8]. However, orthodontic tooth movement may increase the incidence and size of dehiscences in the anterior mandibular region [2, 9]. Dehiscences occur most often in mandibular canine teeth (12.9%) [7, 8]. Fenestrations are more common in the maxilla, but they also occur in the anterior mandible.

Cone-beam computed tomography (CBCT) can be used to detect and measure fenestrations and dehiscences in untreated subjects. In orthodontically treated subjects, CBCT can be used as an extra diagnostic tool, at the beginning and throughout the treatment, to ensure the correct position of the roots of the teeth in the lower anterior mandible. CBCT imaging is attractive, due to its high performance, low cost, and the relatively low doses required, compared to conventional, multi-slice CT [10, 11]. Consequently, the use of CBCT has become increasingly common for diagnostics and treatment planning in dentistry [12]. A recent systematic review has provided evidence for the validity of CBCT in detecting interfurcal, vertical, and horizontal bone losses, which were particularly pronounced in maxillary molars [13]. However, CBCT data should also be validated for diagnostic value in other dental regions.

CBCT can also be used to measure buccal and lingual bone in the anterior mandibular region. However, in this region of the mandible, the bone is often thin. Bone thicknesses less than 0.6 mm increase the risk of false positive diagnoses of dehiscences and fenestrations. CBCT accuracy for measuring bony dehiscences is best when the bone thickness is more than 0.6 mm and the scanning voxel size is 0.250 mm. When CBCTs are performed with a larger voxel size (e.g., 0.400 mm), the size of the dehiscence can be overestimated [14]. Therefore, due to the high density of teeth combined with the thin bone, CBCT lacks accuracy for measuring alveolar bone heights.

Protocols for CBCT imaging involve multiple acquisition settings, including the size of the field of view, exposure time, and voxel size. These parameters influence the image quality and the effective radiation dose. Considering that image quality and effective dose are proportional [15], the best practice is to adhere to the two basic principles of radiation exposure, which recommend using a dose “as low as reasonably achievable” (ALARA) at a resolution “as low as diagnostically acceptable” (ALADA) [16]. Thus, a CBCT protocol with a higher voxel size and lower radiation dose is desirable, when it does not compromise the accuracy in measuring dehiscences and fenestrations in the mandibular anterior region. However, it remains unclear how different voxel sizes affect the accuracy of measuring these defects [14, 17].

The objective of this study was to assess how CBCT scanning protocols with different voxel sizes affected the surface area measurements of dehiscences and fenestrations in the mandibular anterior buccal region. The null hypothesis was that there would be no difference in the surface area measurements of dehiscences and fenestrations in the mandible among CBCT scans performed with three different voxel sizes.

## Materials and methods

### Sample

For this observational study, we selected dry human mandibles from the dry skull collection in the Department of Orthodontics at the University Medical Center Groningen (UMCG), the Netherlands. Selection was based on the following inclusion criteria: intact human mandibles with permanent dentition from one canine to the other canine. Mandibles were excluded when they had obvious pathology, broken or damaged teeth, or dental restorations. A total of 19 mandibles with 114 teeth met the inclusion criteria.

### Surface scans of the mandible

The mandibles were scanned with the Primescan dental intraoral surface scanner (SS) (Dentsply Sirona, New York, NY). Prior to the scanning procedure, the scanner was calibrated according to the manufacturer’s guidelines. All scans were performed by a single operator (BvL). The SS data were converted to the standard tessellation language format. The SS images were used to measure the surface areas of the dehiscences and fenestrations.

### Radiographic procedures

**Fig. 1:**
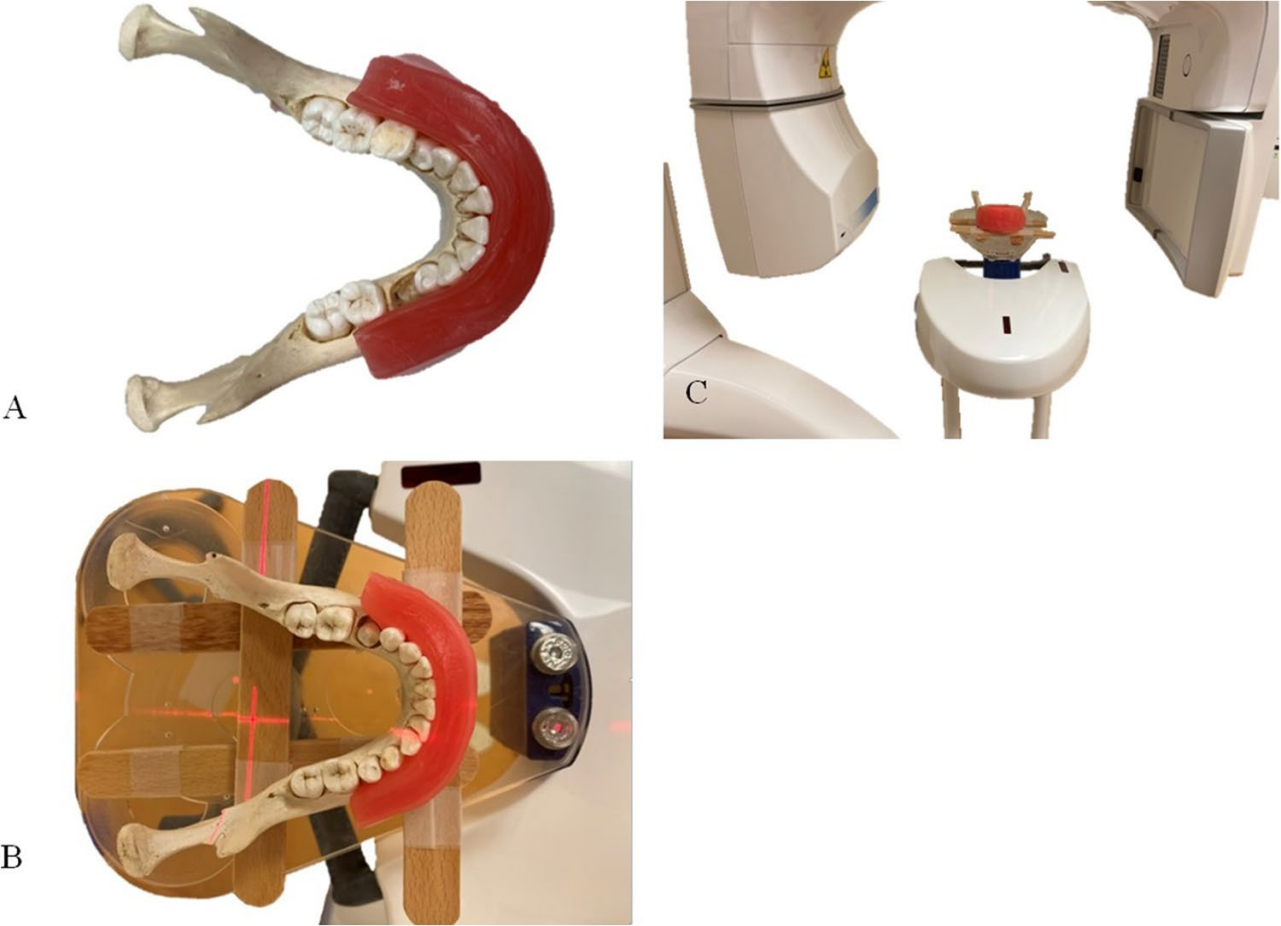
Preparing the mandible for CBCT scanning. **A** Dental modeling wax was attached to the buccal side of the mandible to serve as a soft tissue equivalent. **B**, **C** The mandible was positioned in the center of the scanning table of the CBCT machine, in the same orientation as a live patient would be oriented, aligned with vertical and horizontal laser guides

To simulate soft tissues, dental modeling wax (Set Up Regular, Cavex, Haarlem, Netherlands) was applied to the buccal aspect of each mandible. The wax was spread from the right to left first molars and from the incisal/occlusal edge of the teeth to the inferior border of the mandible (Fig. 1A). The wax was 12 mm thick, based on average soft tissue thickness values [18].

CBCT scans were acquired with a Promax 3D Mid unit (Planmeca, Helsinki, Finland), and they were performed by one operator (BvL). The machine was calibrated prior to collecting the data, according to the manufacturer’s calibration protocol. The mandibles were positioned in the center of the scanning table, in the same orientation as a live patient would be positioned, aligned with vertical and horizontal laser guides (Fig. 1A, B). The field of view was 16.0 × 10.2 cm. Each mandible was scanned three times with three different protocols, including high resolution, 0.200 mm voxel size (VS200), 90 kV, 10.0 mA, and 18.02 s scanning time; mid resolution, 0.400 mm voxel size (VS400), 90 kV, 10.0 mA, and 13.57 s scanning time; and low resolution, 0.600 mm voxel size (VS600), 90 kV, 10.0 mA, and 9.05 s scanning time. Hence, 57 cone-beam scans were performed for 19 mandibles.

The CBCT scan data were exported from Romexis software (version 4.6.1R, Planmeca, Helsinki, Finland) as digital imaging and communications in medicine (DICOM) files and imported into the rendering software, 3D Slicer (version 4.11, Open Source). The raw data were reconstructed into three-dimensional (3D) standard tessellation language files. The 3D surface models of all mandibular images were generated, based on the preset threshold range for bone (250–3071), as specified in the rendering software.

### Measurements

**Fig. 2:**
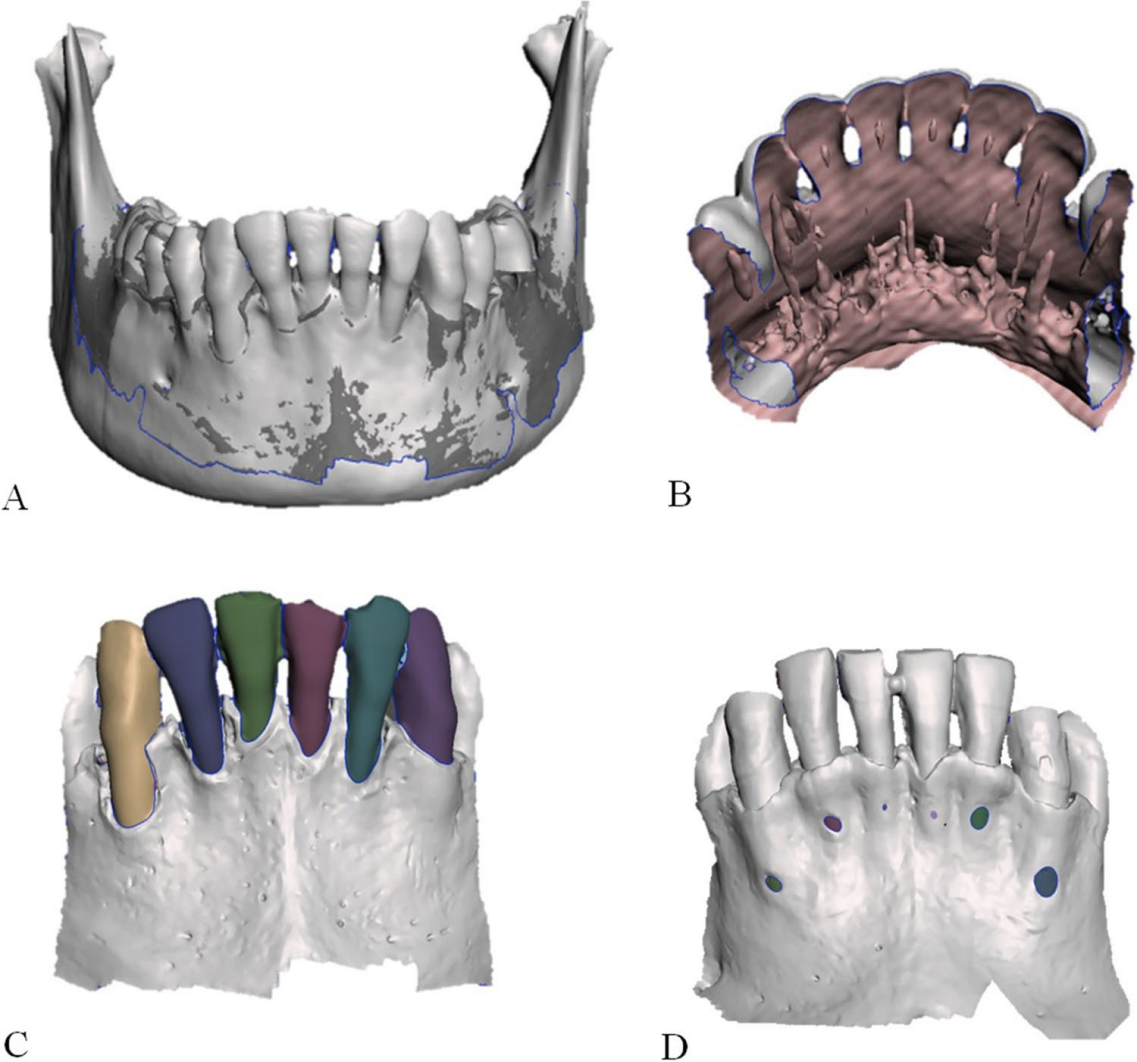
Preparing the datasets for analysis. **A** Anterior view of the three CBCT scans (VS600, VS400, VS200) superimposed on the SS; after alignment, the superimposed images were saved in one dataset. **B** Posterior view of the superimposed dataset (SS, VS200, VS400, VS600), shown after digitally removing the lingual surfaces of the six anterior teeth and the lingual side of the mandibular body. **C** Anterior view shows the buccal surfaces of each of the six anterior teeth; colors show the entire surfaces included in the tooth surface area calculations. **D** An example of a mandible with fenestrations (colored spots) on the buccal side of the mandible. The surface areas of the fenestrations were determined

Surface area measurements of dehiscences and fenestrations were performed with Meshmixer software (version 3.5, Autodesk, San Rafael, CA). The three CBCT scans (VS200, VS400, VS600) and the SS scan of each mandible were imported into the software. The three CBCT scans were all aligned with the SS scan to generate one dataset of the four superimposed scans (Fig. 2A). In this dataset, all lingual surfaces of the six anterior teeth and the lingual side of the mandibular body were removed from the 3D images (Fig. 2B). Thus, the plane of interest ran over the anterior surface, from the incisal and occlusal edge of the teeth, across to the mesial and distal edges, and down to the inferior border of the anterior mandible. The isolation of this plane was performed by a single operator (BvL).

Every scan in each superimposed dataset was viewed separately. The buccal surface area of each tooth (tooth numbers 33 to 43) was determined by marking the perimeter of the tooth area and then using the analysis tool in the Meshmixer software to calculate the surface area (Fig. 2C). The surface areas were measured on 19 mandibles, with 6 teeth each, on 4 separate scans; thus, a total of 456 measurements were performed.

In healthy conditions, the average distance between the cemento-enamel junction (CEJ) and the alveolar crest is 1.5 to 2.0 mm. A dehiscence is the lack of alveolar bone on the buccal or lingual side of a tooth, which results in an exposed cervical root surface. Thus, when a dehiscence occurs, the distance between the CEJ and the alveolar crest is increased. Many authors consider that a dehiscence is present, when this distance is > 2.0 mm [9, 19, 20]. In the present study, a proxy for dehiscence was necessary, because the CEJ is not detectable on CBCT scans. Thus, we could not discern the presence or absence of a dehiscence according to the clinical definition. Instead, we measured the total buccal surface area of the tooth and used it as a proxy of dehiscence. The proxy comprised two fictional parts. The upper part was the buccal surface of the tooth. This surface area ran from the incisal/occlusal edge on top, across to the mesial and distal borders on the sides of the tooth, and down to the imaginary CEJ (Fig. 2C). This perimeter was determined by one observer (BvL) on the superimposed dataset (SS, VS200, VS400, and VS600), by removing the lingual side of the teeth in 3D models. This method ensured that the perimeter of this upper part (incisal/occlusal edge and mesial/distal border of the tooth) was standardized and had the same surface area in the dataset. The lower part was considered the area of dehiscence. It ran from the imaginary CEJ to the alveolar crest. The only remaining source of variation in this method was the assessment of the bony borders.

The surface areas of fenestrations on the buccal side of the mandible were determined with SS by digitally selecting the bone/radix border of the fenestration in the image (Fig. 2D). To determine the gonial angle of the mandible, a virtual cephalogram was reconstructed from the VS200 scan in Romexis software (version 4.6.1R, Planmeca, Helsinki, Finland). Briefly, the mandibular plane was drawn from the menton to the inferior border of the mandible. The ramus plane was drawn from the posterior border of the ramus to the posterior borders of the condyle. Based on these two lines, the gonial angle was determined.

The Little’s Irregularity Index was used to determine the crowding of the six anterior teeth in the mandibular arch [21]. A dial caliper (Schuifmaat 140 mm, Overtoom International, Den Dolder, Netherlands) was used to perform the measurements. The mandibles were categorized into 5 groups, from perfect alignment to very severe irregularity [21].

All measurements (fenestrations, dehiscences, gonial angle, and Little’s Index) were performed in random order by two observers: an orthodontic resident (BvL), with 3 years of experience, and a medical radiation technician (JAD), with 6 years of experience in orthodontic radiology.

### Statistical analysis

Analyses were performed with SPSS (version 26, IBM Corporation, Armonk, NY, USA).

The intraclass correlation coefficient for absolute agreement, based on a two-way random effects model, was used to evaluate the inter-examiner reliability for all measurements.

The statistical analyses were performed with the data from one observer (BvL). The differences between the SS and CBCT measurements were calculated as follows: difference = SS − CBCT. To account for repeated measurements, a linear mixed model analysis (covariance structure auto-regressive 1st order) was performed. The mandible was the highest level on which repeated measurements were performed. The dependent variable was the difference between the SS and CBCT measurements. The potential explaining variables were the CBCT protocol (VS600, VS400, and VS200), the quadrant (left, teeth 33/32/31; right, teeth 43/42/41), the gonial angle, the type of tooth (tooth numbers 31/41, 32/42, and 33/43), the Little Index, and the SS (fixed effect). The fixed effect of the SS was explored, because the difference between the SS and CBCT protocols could be related to the actual SS outcome. All explanatory variables were entered in the model, and thereafter, variables were removed manually, based on the highest *P* value, until all remaining variables were significantly associated with the dependent variable. All steps were verified with the − 2 log likelihood criterion. Thereafter, interaction effects were explored. The level of significance was set at *P* < 0.05.

Agreements between the measurements made with SS and the CBCT measurements were assessed with Bland–Altman plots, with 95% limits of agreement.

## Results

### Reliability

The inter-examiner reliabilities for measurements on SS, VS600, VS400, and VS200 images were excellent. The intraclass correlation coefficient for these 4 scans ranged from 0.989 to 0.997 (Table 1).

**Table 1:** Inter-examiner reliability for dehiscences, calculated for SS, VS600, VS400, and VS200. The intraclass correlation coefficient (ICC) for absolute agreement based on a two-way random effect model was applied

Scan method	ICC	95% CI
SS	0.989	[0.984; 0.992]
VS600	0.996	[0.994; 0.997]
VS400	0.997	[0.996; 0.998]
VS200	0.996	[0.994; 0.997]

### Fenestrations

**Fig. 3:**
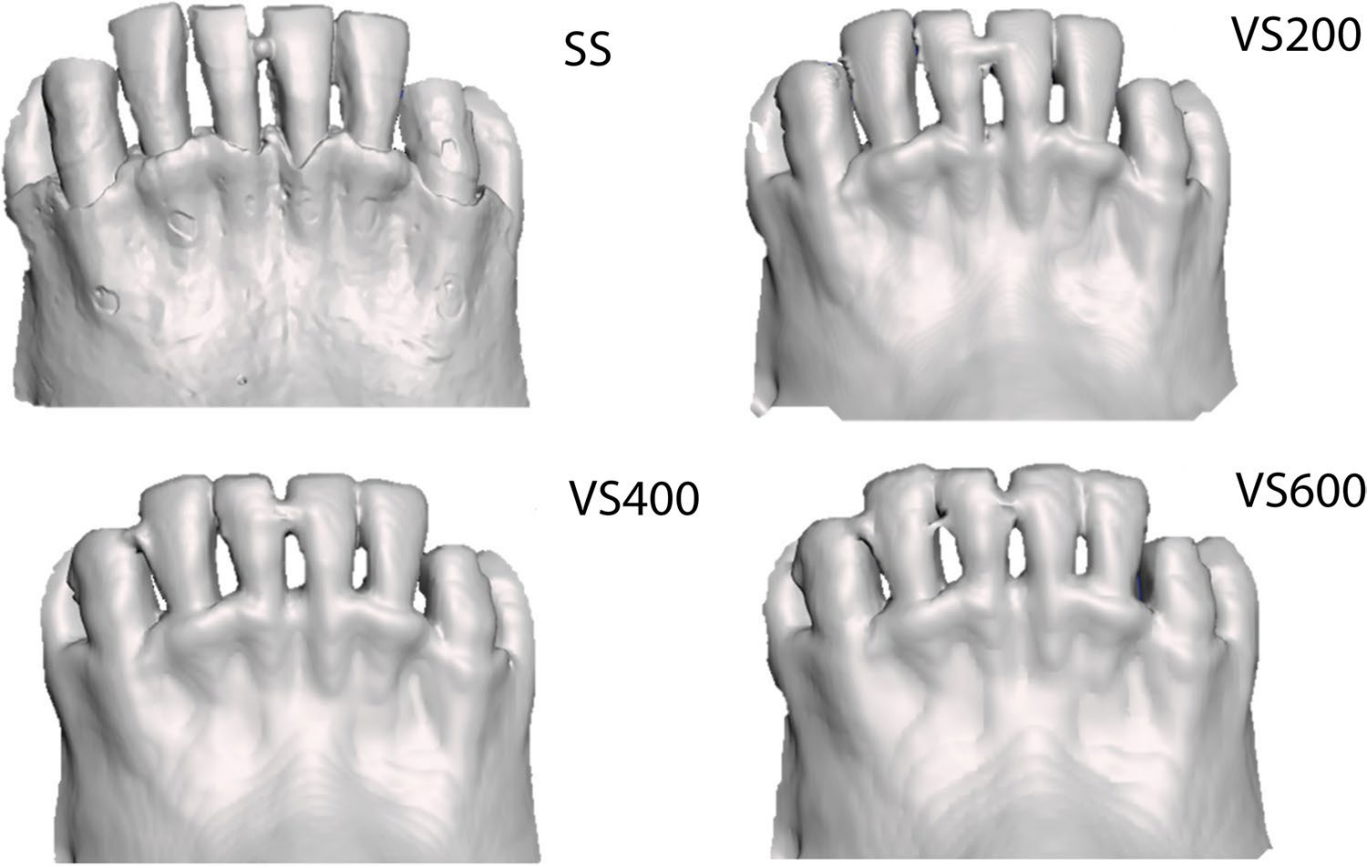
Inability of CBCT to detect fenestrations. (*Top left*) SS image (Pri) of a mandible shows six fenestrations detected by the observers (see Fig. 2D for colored rendition); (*top right and bottom right and left*) CBCT images of the same mandible show that the fenestrations were not detected on CBCT scans with resolutions of VS200 (voxel size 0.200 mm), VS400 (voxel size 0.400 mm), or VS600 (voxel size 0.600 mm)

Twenty-four fenestrations were identified on 11 of the 19 mandibles. None of these fenestrations was detected on the CBCT scans (Fig. 3).

### Dehiscences

In the linear mixed model analysis of dehiscence measurements, the Little Index, the quadrant, the type of tooth, and the gonial angle were removed, in that order, from the model. The CBCT protocol (*P* = 0.029) and the SS fixed effect (< 0.001) were found to influence the difference between the SS and CBCT surface measurements. In the analysis, the difference between the VS600 and SS measurements was designated the reference difference. The difference between the VS200 and SS measurements differed significantly (*P* = 0.009) from the reference difference. The difference between the VS400 and SS measurements did not differ significantly (*P* = 0.067) from the reference difference (Table 2).

**Table 2:** Results of the linear mixed model analyses with the difference between the CBCT measurements and SS (mm^2^) as dependent variable

Variable	Beta	SE beta	*P* value	95% CI
SS	− 0.1	0.0	< 0.001	[− 0.2; − 0.1]
Voxel 600*	2.0	2.0	0.319	[− 2.0; 6.1]
Voxel 400	0.9	0.5	0.067	[− 0.1; 1.9]
Voxel 200	1.3	0.5	0.009	[0.3; 2.3]

^*^Reference category

The mean surface areas per tooth on the CBCT images ranged between 94.6 and 96.0 mm^2^, and they were larger than the mean surface area per tooth on the SS images (88.3 mm^2^). The difference in mean surface areas between the SS and the CBCT images depended on the CBCT protocol. These differences were − 7.7 mm^2^ (8.7%) for the VS600, − 6.8 mm^2^ (7.7%) for the VS400, and − 6.3 mm^2^ (7.0%) for the VS200 protocols (Table 3).

**Table 3:** The measured mean surface area per tooth plus standard deviation and the mean difference (SS–CBCT scan) plus standard deviation (mm^2^)

Image modality	Mean surface area/tooth (mm^2^)	SD (mm^2^)	Mean surface area difference/tooth (mm^2^)	SD (mm^2^)	%
SS	88.3	24.0			
VS600	96.0	28.2	− 7.7	8.9	8.7
VS400	95.1	27.3	− 6.8	7.3	7.7
VS200	94.6	26.5	− 6.3	6.2	7.0

**Fig. 4:**
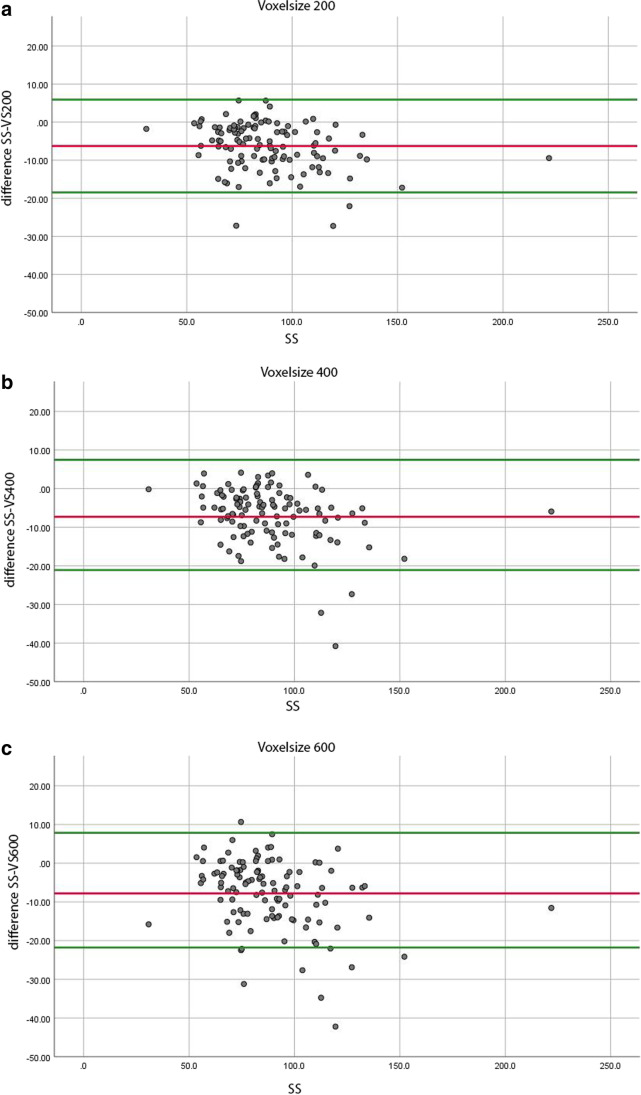
Bland–Altman plots show the degrees of agreement on the dehiscence surface area measurements (mm^2^) between scanner measurements (SS) and CBCT measurements. Dots represent the difference between SS and CBCT measurements. The red line indicates the mean difference between the two imaging results, and the green lines show the 95% limits of agreement. **A** VS200 (voxel size 0.200 mm), **B** VS400 (voxel size 0.400 mm), **C** VS600 (voxel size 0.600 mm)

We constructed Bland–Altman plots to visualize the agreement between the mean surface areas of the digital models (SS) and the differences between the SS and CBCT measurements (Fig. 4). The 95% levels of agreement ranged from − 18.5 to − 5.9mm^2^, for the difference between the SS and the VS200 models (Fig. 4A); − 21.1 mm^2^ to 7.5 mm^2^, for the difference between the SS and VS400 models (Fig. 4B); and − 21.8 mm^2^ to 7.9 mm^2^, for the difference between the SS and VS600 models (Fig. 4C).

**Fig. 5:**
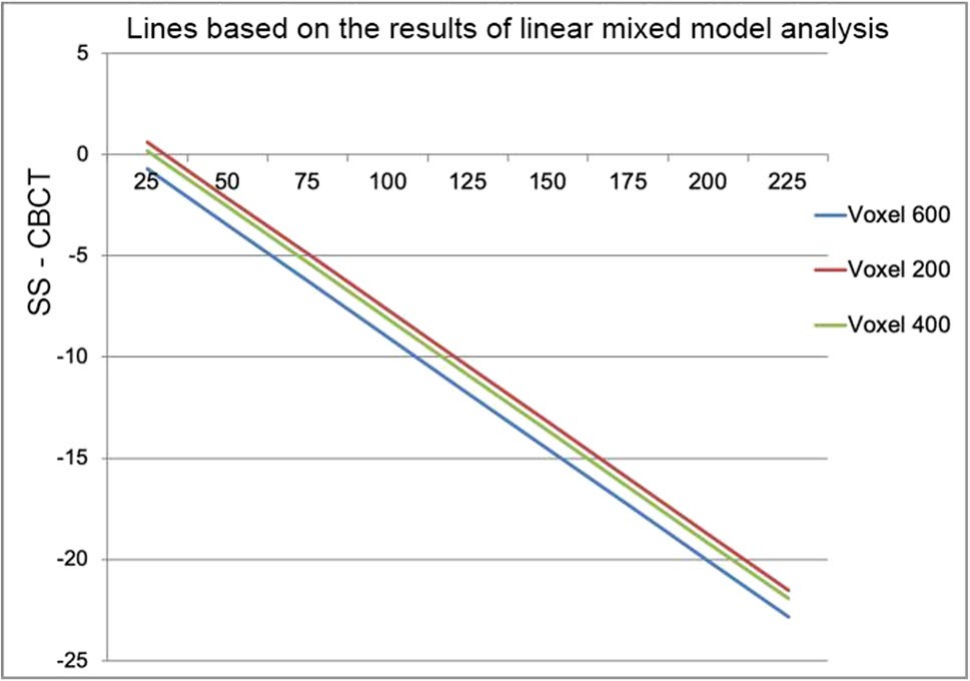
Plot shows that, as the SS surface area measurement increases, the difference between the SS and the CBCT (VS600, VS400, and VS200) surface area measurements increases. The slope is negative, based on the regression coefficients found in the linear mixed model analysis. *X*-axis, SS measurements; *Y*-axis, SS measurement — CBCT measurement

Based on the results of the linear mixed model analysis, we created a graph to illustrate the estimated effects of SS and voxel sizes on the differences in surface area measurements between the SS and CBCT models. Because the regression coefficient of the SS was negative, the lines had negative slopes (Fig. 5).

## Discussion

This study was performed to assess the effect of different CBCT voxel sizes on surface area measurements of fenestrations and dehiscences in the anterior buccal region of the human mandible. With our methods, fenestrations were not detectable on the CBCT scans. However, for dehiscences, the surface areas were larger when calculated on CBCT images than when calculated on SS images. We found that the larger the measured surface area, the larger the difference in mean surface area measurements between the SS and the CBCT methods.

Several studies have endeavored to analyze the accuracy of CBCT in measuring dehiscences and fenestrations. Dry human skulls with natural bony defects have been used previously, but without the use of a soft tissue equivalent [22]. Later studies used cadaver heads [23, 24] to overcome this shortcoming, or they performed the measurements in vivo [25, 26]. Another shortcoming of previous studies was that only the lengths of dehiscences and fenestrations were measured [22–24, 27]. However, length measurements do not accurately represent the true anatomy.

We selected a Primescan dental intraoral SS to produce a digital model of the mandible, due to its high accuracy. A recent study [28] showed that the validity and reliability of this scanner were 25 µm and 10 µm, respectively.

CBCT scans were acquired with a Promax 3D Mid unit with three different protocols. Therefore, the results do not necessarily apply to different CBCT units and protocols. 3D digital models of the CBCT scans were derived from semi-automatic segmentation (3D Slicer), which is the clinical standard nowadays [29]. Because CBCT data has an intrinsic low image contrast, lack of Hounsfield units, and increased noise and artifacts compared to multi-slice CT, semi-automatic segmentation requires manual edits which could influence the reliability. Further developments with regard to fully automatic segmentation with artificial intelligence (AI) models could solve this shortcoming [30, 31].

A soft tissue equivalent was added to the human mandibles, because the surrounding structures can influence the gray values of CBCT scans [32]. The absence of soft tissue can lead to false positive detection of bony defects [23]. A soft tissue equivalent must have a pixel intensity similar to that of human soft tissue. A recent study [33] explored the effect of different soft tissue equivalents (ice, modeling wax, ballistic gelatin) on the pixel intensity of bone and tooth structures surrounded by these substances. Piglet heads with intact soft tissue was used as the gold standard. Ballistic gelatin was found to be the best soft tissue equivalent in the mandible, and it was closely followed by modeling wax. These two simulants had similar influences on the pixel intensities of the bone and the teeth. We selected modeling wax, because we wanted to avoid permanent damage to the dry skull mandibles, which is caused by the physical characteristics of ballistic gelatin.

Although we attached a 12-mm layer of wax to the buccal side of the mandible, this surface only partially simulated soft tissue. Ideally, human cadaver heads or skulls completely filled with a soft tissue simulant would give the best, most realistic representation, because CBCT derives from projections obtained at multiple angles around the object. However, filling the dry skulls with wax was not possible in our setting.

On CBCT scans, the surface areas were enlarged compared to the areas measured with digital models of the mandible. We found that the larger the measured surface area of a tooth, the larger this enlargement with CBCT (Fig. 5). In VS600 images, the surface areas were the largest, and in VS200 images, the surface areas were the smallest. CBCT measurements were most accurate, when the bone thickness was > 0.6 mm [14]. Thin bone was easier to detect with the smaller voxel size, which provided higher resolution. With a larger voxel size, thin buccal bone might be undetectable; thus, the dehiscence might appear larger than its actually size (Table 3). The chance of a false positive detection of dehiscence increased with the size of the surface area.

Our results were consistent with results from older studies [22–24, 27] that reported improved accuracy with smaller voxel sizes. We found small differences in surface areas measured with the different voxel sizes and the surface areas measured on the digital images (Table 3). Thus, the three CBCT protocols were accurate for measuring dehiscences. However, the percentage differences between the scans would have been larger, if we had measured the real dehiscence, instead of using the entire buccal surface area (Table 3).

We also found that the limits of agreement between the SS and CBCT models were widest in the lowest CBCT resolution group. Thus, the lower the CBCT resolution, the less reliability in detecting dehiscences. However, these differences were small and not clinically relevant (Fig. 4).

The resolution should be selected, based on the accuracy of the CBCT protocol, the degree of reliability considered clinically acceptable, and the radiation dose. This decision should be made on a case to case basis. When clinical dehiscence with thin buccal bone coverage is expected, a CBCT scan could be used as an extra diagnostic tool to detect this defect. To minimize the chance of a false positive detection of this bony defect, we recommend a CBCT protocol with a small field of view and high resolution (VS200). When a low resolution CBCT scan has been performed for other diagnostic reasons, and there is no clinical indication of a dehiscence, an additional high resolution scan is not needed.

In this study, bony fenestrations were not detected with the CBCT protocols. This finding was expected, because the bone around a fenestration is thin, and CBCT cannot detect thin bony defects CBCT [14].

During in vivo scanning, head movement can reduce the scanning accuracy. In the present study, no movement of the mandibles occurred during CBCT imaging. Therefore, our results were probably more accurate than might be expected with in vivo scanning.

## Conclusion

This study showed that CBCT imaging magnified the surface areas of dehiscences in the lower buccal anterior region of the mandible. We found that the lower the CBCT scan resolution, the larger the deviation of CBCT measurements relative to measurements performed on digital models of the mandible. However, the differences were small. Therefore, our findings indicated that CBCT protocols with voxel sizes of 0.600 mm, 0.400 mm, and 0.200 mm were acceptable for measuring dehiscences. However, it should be noted that the chance of a false positive detection of a dehiscence increased for larger surface areas. Fenestrations were not detectable with this method.

## References

[1] Wehrbein H, Bauer W, Diedrich P (1996). Mandibular incisors alveolar bone and symphysis after orthodontic treatment A retrospective study. Am J Orthod Dentofacial Orthop.

[2] Sheng Y, Guo HM, Bai YX, Li S (2020). Dehiscence and fenestration in anterior teeth : comparison before and after orthodontic treatment. J Orofac Orthop.

[3] Artun J, Krogstad O (1987). Periodontal status of mandibular incisors following excessive proclination A study in adults with surgically treated mandibular prognathism. Am J Orthod Dentofacial Orthop.

[4] Yared KF, Zenobio EG, Pacheco W (2006). Periodontal status of mandibular central incisors after orthodontic proclination in adults. Am J Orthod Dentofacial Orthop.

[5] Wennström JL, Lindhe J, Sinclair F, Thilander B (1987). Some periodontal tissue reactions to orthodontic tooth movement in monkeys. J Clin Periodontol.

[6] Melsen B, Allais D (2005). Factors of importance for the development of dehiscences during labial movement of mandibular incisors: a retrospective study of adult orthodontic patients. Am J Orthod Dentofacial Orthop.

[7] Nimigean VR, Nimigean V, Bencze MA, Dimcevici-Poesina N, Cergan R, Moraru S (2009). Alveolar bone dehiscences and fenestrations: an anatomical study and review. Rom J Morphol Embryol.

[8] Rupprecht RD, Horning GM, Nicoll BK, Cohen ME (2001). Prevalence of dehiscences and fenestrations in modern American skulls. J Periodontol.

[9] Evangelista K, de Faria Vasconcelos K, Bumann A, Hirsch E, Nitka M, Silva MA (2010). Dehiscence and fenestration in patients with class I and class II division 1 malocclusion assessed with cone-beam computed tomography. Am J Orthod Dentofacial Orthop.

[10] Dula K, Benic GI, Bornstein M, Dagassan-Berndt D, Filippi A, Hicklin S, Kissling-Jeger F, Luebbers HT, Sculean A, Sequeira-Byron P, Walter C, Zehnder M (2015). SADMFR guidelines for the use of cone-beam computed tomography/digital volume tomography. Swiss Dent J.

[11] Jaju PP, Jaju SP (2015). Cone-beam computed tomography: time to move from ALARA to ALADA. Imaging Sci Dent.

[12] Kaeppler G (2010). Applications of cone beam computed tomography in dental and oral medicine. Int J Comput Dent.

[13] Walter C, Schmidt JC, Rinne CA, Mendes S, Dula K, Sculean A (2020). Cone beam computed tomography (CBCT) for diagnosis and treatment planning in periodontology: systematic review update. Clin Oral Investig.

[14] Rédua RB, Carvalho FAR, Artese FRG (2020). Measurement of the bone height of mandibular incisors and canines on computed tomography-limitations according to bone thickness. Orthod Craniofac Res.

[15] Palomo JM, Rao PS, Hans MG (2008). Influence of CBCT exposure conditions on radiation dose. Oral Surg Oral Med Oral Pathol Oral Radiol Endod.

[16] Yeung AWK, Jacobs R, Bornstein MM (2019). Novel low-dose protocols using cone beam computed tomography in dental medicine: a review focusing on indications, limitations, and future possibilities. Clin Oral Investig.

[17] Evans M, Tanna NK, Chung C-H (2016). 3D guided comprehensive approach to mucogingival problems in orthodontics. Seminars in Orthodontics.

[18] Kotrashetti VS, Mallapur MD (2016). Radiographic assessment of facial soft tissue thickness in South Indian population–an anthropologic study. J Forensic Leg Med.

[19] Jäger F, Mah JK, Bumann A (2017). Peridental bone changes after orthodontic tooth movement with fixed appliances: a cone-beam computed tomographic study. Angle Orthod.

[20] Yagci A, Veli I, Uysal T, Ucar FI, Ozer T, Enhos S (2012). Dehiscence and fenestration in skeletal class I, II, and III malocclusions assessed with cone-beam computed tomography. Angle Orthod.

[21] Little RM (1975). The irregularity index: a quantitative score of mandibular anterior alignment. Am J Orthod.

[22] Leung CC, Palomo L, Griffith R, Hans MG (2010). Accuracy and reliability of cone-beam computed tomography for measuring alveolar bone height and detecting bony dehiscences and fenestrations. Am J Orthod Dentofacial Orthop.

[23] Damstra J, Fourie Z, Huddleston Slater JJ, Ren Y (2010). Accuracy of linear measurements from cone-beam computed tomography-derived surface models of different voxel sizes. Am J Orthod Dentofacial Orthop.

[24] Patcas R, Müller L, Ullrich O, Peltomäki T (2012). Accuracy of cone-beam computed tomography at different resolutions assessed on the bony covering of the mandibular anterior teeth. Am J Orthod Dentofacial Orthop.

[25] Peterson AG, Wang M, Gonzalez S, Covell DA, Katancik J, Sehgal HS (2018). An in vivo and cone beam computed tomography investigation of the accuracy in measuring alveolar bone height and detecting dehiscence and fenestration defects. Int J Oral Maxillofac Implants.

[26] Sun L, Zhang L, Shen G, Wang B, Fang B (2015). Accuracy of cone-beam computed tomography in detecting alveolar bone dehiscences and fenestrations. Am J Orthod Dentofacial Orthop.

[27] Cook VC, Timock AM, Crowe JJ, Wang M, Covell DA (2015). Accuracy of alveolar bone measurements from cone beam computed tomography acquired using varying settings. Orthod Craniofac Res.

[28] Diker B, Tak Ö (2020). Comparing the accuracy of six intraoral scanners on prepared teeth and effect of scanning sequence. J Adv Prosthodont.

[29] Fan Y, Beare R, Matthews H, Schneider P, Kilpatrick N, Clement J, Claes P, Penington A, Adamson C (2019). Marker-based watershed transform method for fully automatic mandibular segmentation from CBCT images. Dentomaxillofac Radiol.

[30] Verhelst PJ, Smolders A, Beznik T, Meewis J, Vandemeulebroucke A, Shaheen E, Van Gerven A, Willems H, Politis C, Jacobs R (2021). Layered deep learning for automatic mandibular segmentation in cone-beam computed tomography. J Dent.

[31] Wang G, Li W, Zuluaga MA, Pratt R, Patel PA, Aertsen M, Doel T, David AL, Deprest J, Ourselin S, Vercauteren T (2018). Interactive medical image segmentation using deep learning with image-specific fine tuning. IEEE Trans Med Imaging.

[32] Molteni R (2013). Prospects and challenges of rendering tissue density in Hounsfield units for cone beam computed tomography. Oral Surg Oral Med Oral Pathol Oral Radiol.

[33] Lopes PA, Santaella GM, Lima CAS, Vasconcelos KF, Groppo FC (2019). Evaluation of soft tissues simulant materials in cone beam computed tomography. Dentomaxillofac Radiol.

